# Pop Goes the Lung: Bilateral Pneumothoraces Due to Delayed Pneumatocele Rupture in Coronavirus Disease 2019 (COVID-19)

**DOI:** 10.7759/cureus.52008

**Published:** 2024-01-10

**Authors:** Bashar Khiatah, Amanda Frugoli, Ralph Akl, Allan Wagner, Brian Utz, Robert Bernstein

**Affiliations:** 1 Internal Medicine, Overlake Medical Center, Bellevue, USA; 2 Graduate Medical Education, Internal Medicine, Community Memorial Hospital, Ventura, USA; 3 Family Medicine, Community Memorial Hospital, Ventura, USA; 4 Pulmonary Medicine, Community Memorial Hospital, Ventura, USA

**Keywords:** covid-associated pneumothorax, covid-19, bilateral spontaneous pneumothorax, covid-19 complication, pneumatocele

## Abstract

Worldwide medical and scientific communities are focusing on further understanding coronavirus disease 2019 (COVID-19) complications and its long-term impact on survivors. Pneumatocele cases are being reported more as a consequence of this virus and a cause of pneumothorax in certain patients. In this case vignette, we present a previously healthy male with COVID-19 symptoms who required hospitalization for hypoxia and who required readmission for bilateral pneumothorax from the delayed rupture of pneumatoceles. We describe this rare pathology and provide hypotheses for possible etiologies.

## Introduction

Spontaneous pneumothorax has been reported as a complication of severe acute respiratory syndrome coronavirus 2 (SARS-CoV-2) (coronavirus disease 2019 (COVID-19)) and is largely reported as case series or small retrospective studies. The initial estimates for pneumothorax development in COVID-19 were 1% of patients requiring hospital admission and 2% of those requiring intensive care unit (ICU) admission, but some more recent studies suggest it may be seen in up to 8% of patients [1,2]. There is less information regarding the development of pneumothorax in patients with COVID-19 who are managed in the outpatient setting. Ongoing research into the underlying pathophysiology is underway, but there may be multiple etiologies for this complication.

Pneumatocele causing pneumothorax, pneumopericardium, and pneumomediastinum has been reported as a complication of COVID-19 infection [3,4]. COVID-19 can cause pneumatocele via multiple etiologies including direct alveolar destruction by the virus, multiple pulmonary embolism at a microvascular level causing necrotizing of the pulmonary tissues, and an inflammatory reaction causing thickening of the bronchial wall and thick mucus forming an endobronchial valve [5-9].

In the era of COVID-19, more cases of pneumatocele are being reported during the acute illness phase. In this vignette, we describe a healthy 36-year-old male who presented with COVID-19 symptoms and hypoxia requiring hospitalization and treatment with dexamethasone plus remdesivir and who was discharged without complications but presented later with worsening symptoms due to bilateral pneumothorax caused by the rupture of pneumatoceles. 

## Case presentation

**Figure 1:**
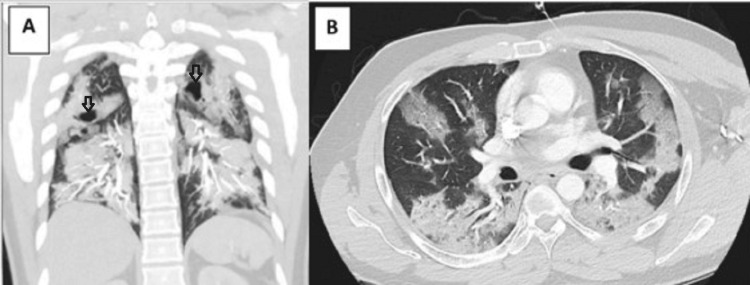
Coronal and axial CT imaging showing extensive consolidation and ground-glass opacities bilaterally with pneumatoceles (arrows) A: Coronal CT demonstrating extensive consolidation and bilateral pneumatoceles (arrows) B: Axial CT showing extensive multi-lobar consolidation with ground-glass opacities CT: computed tomography

A 36-year-old healthy male presented to the emergency room for progressive shortness of breath and cough over the past six days associated with dysgeusia, anosmia, and diarrhea. He had no known underlying lung disease, tobacco exposure, or occupational exposures. His COVID-19 polymerase chain reaction (PCR) was positive, and his initial computed tomography (CT) angiogram demonstrated extensive bilateral, multi-lobar pneumonia with evidence of pneumatoceles (Figure 1). He was hypoxic with tachypnea but no evidence of respiratory distress. 

He was treated in the hospital for five days with supplemental high-flow oxygen, dexamethasone, and remdesivir and was able to be discharged home. Eight days later, he followed up in the clinic for ongoing shortness of breath and hemoptysis. His vitals demonstrated redevelopment of hypoxia with significant tachycardia. He was sent for an urgent CT angiogram that demonstrated bilateral pneumothorax with ongoing but improved multi-lobar consolidations (Video 1).

**Video 1:** Axial CT images demonstrating bilateral pneumothorax and extensive multi-lobar consolidations with ground-glass opacities CT: computed tomography

He underwent bilateral CT-guided chest tube placement with bilateral lung re-expansion. By hospital day 5, chest tubes had been removed, and he was oxygenating well on room air with minimal dyspnea on exertion. Subsequent imaging demonstrated near resolution of pneumothoraces and bilateral pneumatoceles, and he was able to be discharged home. On an outpatient follow-up one week later, he was well and without respiratory symptoms. 

## Discussion

Pneumatoceles are defined as acquired, thin-walled, intraparenchymal, and air-containing lesions in the lungs [4]. They represent pulmonary tissue without discrete epithelial wall. As patients with COVID-19 are having increased radiographic surveillance, pneumatoceles are being found incidentally or as a result of a significant illness [9,10]. Although some pneumatoceles can be identified on routine chest radiographs, CT chest imaging would have improved sensitivity for detecting small pneumatoceles and evaluating the surrounding lung for consolidation and ground-glass opacities [10].

It is possible that pneumothorax that results during COVID-19 can be related to the development and spontaneous rupture of otherwise clinically insignificant pneumatoceles. There are multiple case reports of lung pathology identified on imaging, like the case report by Jamal et al., who described a large pneumatocele without rupture in a young otherwise healthy adult with COVID-19 that elutes with time [10]. This is in contrast to Pyae and Arif, who described a similar case of a 64-year-old with COVID-19 complicated by pulmonary embolism that has delayed pneumothorax from pneumatocele rupture 20 days after hospitalization [11]. Our case report is similar to Natarajan et al., who described a 32-year-old firefighter who also developed bilateral pneumothoraces with evidence of pneumatoceles and secondary bacterial infection [12]. Our case is dissimilar as our patient had less severe illness, had expeditious recovery, and did not receive anti-IL-6 treatment. 

There have been few retrospective studies with conflicting results correlating pneumothorax to higher mortality and illness severity [13]. One of the earliest retrospective reviews by Chopra et al., evaluating pneumothoraces' outcomes, suggested worsening underlying lung disease and higher mortality [14]. In a different retrospective review, Martinelli et al. agreed pneumothorax is a known complication of COVID-19 but was not an independent risk factor for mortality [9]. Publications this year, by Geraci et al., also concur that pneumothorax is associated with increased mortality and patients treated with large-bore chest tubes may have fewer complications [15]. Dar et al. evaluated the individual risk factors for pneumothorax development in COVID-19 [16]. In this research, they identified two major risk factors of coronary artery disease and mode of oxygen delivery [16].

It is also possible that the development of pneumatoceles represents evidence of pneumatocyte destruction. The pathophysiology of pneumatoceles remains unclear despite multiple theories postulating pulmonary infarction as a possible cause for cavitation. Another hypothesis includes barotrauma which would primarily affect patients on mechanical ventilation and noninvasive positive pressure support measures [9]. The endobronchial check valve is another hypothesis of pneumatocele etiology that was consistently reported in the literature that allows air trapping during inhalation and the formation of a cyst distal to the obstruction caused by inflammation and thickened mucus [8]. The duration and the severity of the blockage determine the cyst size which could reach a massive size rupturing later on and causing pneumothorax. Direct viral destruction of the lung parenchyma or destruction from the activated immune system may also explain the pneumatocele formation as this would correlate to prior cases and studies associating pneumatocele and pneumothorax with disease severity.

Treatment of pneumothorax depends on patient stability and the presence of underlying lung disease [17]. Primary pneumothorax occurs when there is no overt underlying lung disease, while secondary pneumothorax usually occurs in the context of a known disease. Clinically unstable patients should have urgent needle or catheter decompression with catheter size ranging from 16F to 22F [18]. In contrast, clinically stable patients with a small pneumothorax less than 3 cm from the apex to cupola could be treated with conservative management and serial radiographs to confirm stability. They require close follow-up to verify reabsorption [17,18]. Initiation of high-flow oxygen to create a nitrous oxide gradient and aid in reabsorption is considered for all patients.

There are no specific studies that evaluate the risk of the development of long-term lung disease such as fibrosis, obstructive lung disease, or malignancy based on COVID-19 variant or treatment received. Additionally, there is no specific study that correlates COVID-19-related pneumothorax to long-term pulmonary outcome or risk of reoccurrence. One prospective study from the United Kingdom Interstitial Lung Disease (UKILD) Consortium was able to estimate that the prevalence of residual abnormalities in patients that were hospitalized for COVID-19 was about 11% [19]. This study utilized chest radiograph for the comparison that may miss fibrotic lung disease if the diffusing capacity of the lungs for carbon monoxide (DLco) was not affected [19]. Additionally, the current knowledge base is unable to determine time to recovery or future complication. It is also possible that nuances in the imaging may be able to predict the risk of developing lung disease and determine if there is increased risk for recurrence.

## Conclusions

Many etiologies have been reported to cause pneumatocele in patients with COVID-19. We hypothesize that our patient's complication of bilateral pneumothoraces is related to the spontaneous rupture of pneumatoceles that developed as sequelae of COVID-19 infection. Physicians should be cognizant that pneumothorax can occur late in the disease progression or after clinical improvement. Further studies are needed to investigate pneumatocele and pneumothorax development in COVID-19 to determine the underlying pathophysiology and modifiable risk factors and correlate this complication to short- and long-term outcomes.
